# Special Issue “Human Papillomavirus Clinical Research: From Infection to Cancer”

**DOI:** 10.3390/jcm11144225

**Published:** 2022-07-21

**Authors:** Steven F. Gameiro, Joe S. Mymryk

**Affiliations:** 1 McMaster Immunology Research Centre, Department of Medicine, McMaster University, Hamilton, ON L8S 4K1, Canada; 2 Department of Microbiology & Immunology, Oncology and Otolaryngology, Head & Neck Surgery, The Western University, London, ON N6A 3K7, Canada; jmymryk@uwo.ca; 3 London Regional Cancer Program, Lawson Health Research Institute, London, ON N6C 2R5, Canada

Papillomaviruses (PVs) are ubiquitous intracellular pathogens that have co-evolved with many different species. Regardless of species, infection with PVs typically induces benign hyperplastic growths of epithelial cells [1]. In humans, there are over 400 identified human papillomavirus (HPV) types that have an exclusive tropism for either cutaneous or mucosal epithelia [2]. The mucosa-associated types are some of the most researched viruses in the world, due to their established carcinogenic potential. Indeed, the World Health Organization’s (WHO’s) International Agency for Research on Cancer (IARC) have designated 12 HPV types as potent biological carcinogens due to their collective role in causing an estimated 4.1% of the global cancer burden [3,4]. Worldwide, HPVs are the most common sexually transmitted viral infection and because of their highly transmissible nature, most sexually active individuals will become infected at some point throughout their lifetime [5]. HPVs sexual mode of transmission allows it to infect several different mucosal tissues, where most infections will likely be asymptomatic and self-resolving; however, some can potentially lead to the development of cancer at different sites that include the cervix, penis, other anogenital sites, and the head and neck region [6].

According to the most recent global statistics of new cancers, in 2018, HPV was estimated to be responsible for virtually all cases of cervical and anal cancers, a significant fraction of vaginal (78%), vulvar (25%), penile (53%), and an increasing number of head and neck cancers (HNC; 34%)—inclusive of the oropharynx (30%), oral cavity (2%), and larynx subsites (2%) [3]. Importantly, the percentage of these different HPV-induced cancers varies by geographical region and level of economic development. This is evidenced by head and neck cancers and other anogenital cancers being highest in high-income countries, and inversely, cervical cancers being highest in low-income countries [3,7]. This disproportionate variation is partly due to the lack of access to preventative HPV vaccination programs and cervical screening initiatives in low-income countries that have been extensively implemented in high-income economies [3,8].

Pioneering research conducted in the early 90s demonstrated the ability of recombinant L1, HPVs major capsid protein, to self-assemble into virus-like particles (VLPs). These VLPs were type-restrictive in their ability to induce neutralizing antibodies, and therefore multiple VLPs generated from the major wart-inducing and carcinogenic types were necessary to achieve broad protection [6]. The availability of these vaccines will have a major impact in reducing the prevalence of HPV-induced cancers in countries that have successfully implemented vaccination programs against these epitheliotropic viruses. However, many low- to middle-income countries do not have the resources to implement these programs and there are many infections caused by HPV types that these vaccines will not protect against that could develop into cancer [3,8]. In addition, these vaccines are not therapeutic; they are prophylactic, meaning that they will not benefit the millions of people worldwide with existing HPV infections. Consequently, despite the availability of 3 commercial vaccines, HPV will remain an important human pathogen and a major cause of cancer, and therefore will continue to be extensively studied in the long term. Interestingly, this situation is akin to the hepatitis B virus, despite the widespread availability of a commercial vaccine, since 1986, it remains a major health threat for humans [9].

An exciting, and hot area, of clinical research on HPV-induced cancers is the revelation of distinct differences compared to anatomically-similar HPV-negative (HPV^−^) counterparts that has implications for diagnosis, treatment, and outcomes. In recent years, many clinical research initiatives, specifically in the head and neck region, have profiled these differences at the epidemiological, molecular, immunological, and clinical levels. Indeed, in the context of HNCs, the HPV^+^ subtype has more immune cell infiltration into the local tumor microenvironment, greater incidence of T-cell activation, and higher levels of immunoregulatory stimuli compared to HPV^−^ HNCs [10,11,12]. Interestingly, there is evidence that the same immunological differences could hold true for HPV^+^ and HPV^−^ cervical cancers [13]; however, this area of research seems to be limited, most likely due to difficulties of obtaining HPV^−^ cervical cancer samples since virtually all cervical cancers are caused by this sexually transmitted virus.

These distinctive immunological features observed in HPV^+^ HNC may be due to the continuous expression of the viral E6 and E7 oncoproteins within the local tumor microenvironment that initiate an immune response. In addition, patients with HPV^+^ HNC also have greater sensitivity to chemotherapy and radiotherapy compared to those with the HPV^−^ subtype, leading to a more favorable prognosis, with lower recurrence rate, and longer overall survival time [14,15]. This has initiated several clinical trials to evaluate the de-escalation of current therapeutic protocols for the management of patients with HPV^+^ HNC, with the goal of sparing individuals from high-grade toxicities induced by aggressive radiation and chemotherapies that were originally developed for the treatment of individuals with the HPV^−^ subtype of cancer [16,17]. The improved response to treatment and superior clinical outcomes in patients with HPV^+^ HNC could be due to the differences in the immune landscape of these anatomically identical carcinomas with very different etiologies. Intriguingly, prognosis for patients with HPV^+^ cervical cancer is also better than their HPV^−^ counterparts, supporting the presence of parallel processes of treatment response in completely separate anatomical sites [18,19,20].

An abundance of clinical research has provided clear evidence that immune checkpoints, negative regulators of the immune response, are upregulated in HPV^+^ HNC. Indeed, these tumor microenvironments have higher levels of LAG3, PD1, TIGIT, TIM3, and VISTA compared to their HPV^−^ counterparts [11,21,22,23]. This phenomenon has allowed for the potential of novel treatment options since many of these upregulated immune checkpoints are targets of current clinically approved inhibitors for other cancers. Notably, a recent meta-analysis illustrated that those patients with HPV^+^ HNC, with high levels of PD1 expression, displayed improved outcomes when treated with PD1 inhibitors compared to those patients with the HPV^−^ subtype [24]. This demonstration of improved response to PD1 checkpoint inhibitors in clinical trials suggests that HPV^+^ HNCs may similarly respond to other immune checkpoint inhibitors, which has implications for de-intensification from the highly toxic traditional therapies and the possible reduction of treatment-induced sequelae.

From the explicit demonstration of the viral origins and contagious nature of warts in the 20s, to the ground-breaking discovery of select HPV types as the causative agents of cervical cancer and a subset of head and neck cancers in the ‘80s and early 2000s, respectively. Clinical research on this important human pathogen has continuously shaped the field and pushed the boundaries of our collective knowledge. Today, it is well-established that infection with certain types of HPVs can give rise to cervical cancers, other anogenital cancers, and HNCs—one of the fastest rising cancers in some countries despite the availability of effective vaccines. The culmination of nearly a century of clinical research initiatives have established certain HPV types as potent human biological carcinogens, collectively responsible for an estimated 4.1% of the global cancer burden that are distinct from their HPV^−^ counterparts from an epidemiological, molecular, immunological, and clinical perspective. For this Special Issue, we invite authors working in the field to submit state-of-the-art original research and comprehensive reviews that encompass the current landscape of human papillomavirus clinical research, from infection to cancer. Although the primary focus is on infection and cancer of the head and neck region, we will consider HPV relevant manuscripts from other anatomical disease sites.
